# Care Facilities for Older People With Long-Term Substance Use: Promising Practices From Sweden

**DOI:** 10.1093/geroni/igab046.871

**Published:** 2021-12-17

**Authors:** Tove Harnett, Hakan Jonson

**Affiliations:** 1 Lund University, Lund, Skane Lan, Sweden; 2 Lund University, Lund University, Skane Lan, Sweden

## Abstract

The stigma of alcohol and long-term substance use is well-known and may be even greater for older people. This is a presentation on “wet” eldercare facilities, i.e. care settings designed for older people with long-term substance use problems, where abstinence is abandoned for well-being. Wet eldercare facilities exist in several European countries and the Swedish ones have a hybrid formal organization: They target people over 50 years, but are regarded as nursing homes and residents lease their own flats inside the setting, which makes it correct to describe residents as tenants. Guided by symbolic interactionism, the aim is to analyze how residents in wet eldercare facilities manage to view these places in a positive light. Forty-two residents of four facilities were interviewed, revealing how the hybrid status of these places enabled residents to frame their situation as being “in the right place”, but for different reasons. Some framed the place as a nursing home, others as an ordinary flat. Although wet eldercare facilities are undisputedly linked to stigma and the inability to become sober, the formal hybrid organization enabled residents to construct less stigmatized characterizations of the place and of themselves. The study suggests that it is an (often-neglected) gerontological responsibility to counter stigma and improve the sense of dignity for older people living in stigmatized settings. Based on promising practices in the Swedish system, the study therefore presents strategies that enable older people to ascribe positive characteristics to themselves and to the place where they live.

